# [Corrigendum] Role of Smad3 signaling in the epithelial-mesenchymal transition of the lens epithelium following injury

**DOI:** 10.3892/ijmm.2026.5748

**Published:** 2026-01-29

**Authors:** Fanlan Meng, Jun Li, Xiao Yang, Xiaoyong Yuan, Xin Tang

Int J Mol Med 42: 851-860, 2018; DOI: 10.3892/ijmm.2018.3662

Subsequently to the publication of the above paper, an interested reader drew to the authors' attention that, concerning the immunofluorescence images shown in Fig. 2C on p. 855, the 'Blank/E-cadherin' and 'TGF-β2-SIS3/E-cadherin' data panels appeared to show the same data, albeit with different intensities of staining. In addition, in Fig. 3B on p. 856, the GAPDH blots shown for the '7 days' and '28 days' experiment gels were strikingly similar in appearance, in spite of different experiments being reported.

After having asked the authors to explain the apparent anomalies in these figures, they realized that they had been assembled erroneously. Corrected versions of Figs. 2 and 3, now showing the correct data for the 'TGF-β2-SIS3/E-cadherin' experiment in Fig. 2C and the GAPDH western blots for the '28 days' experiment in Fig. 3B, are shown opposite and on the next page. The errors made in assembling Figs. 2 and 3 did not grossly affect either the results or the conclusions reported in this paper. All the authors agree with the publication of this corrigendum, and are grateful to the Editor of *International Journal of Molecular Medicine* for allowing them the opportunity to present this; moreover, the Editor and the authors apologize to the readership for any inconvenience caused.

## Figures and Tables

**Figure 2:**
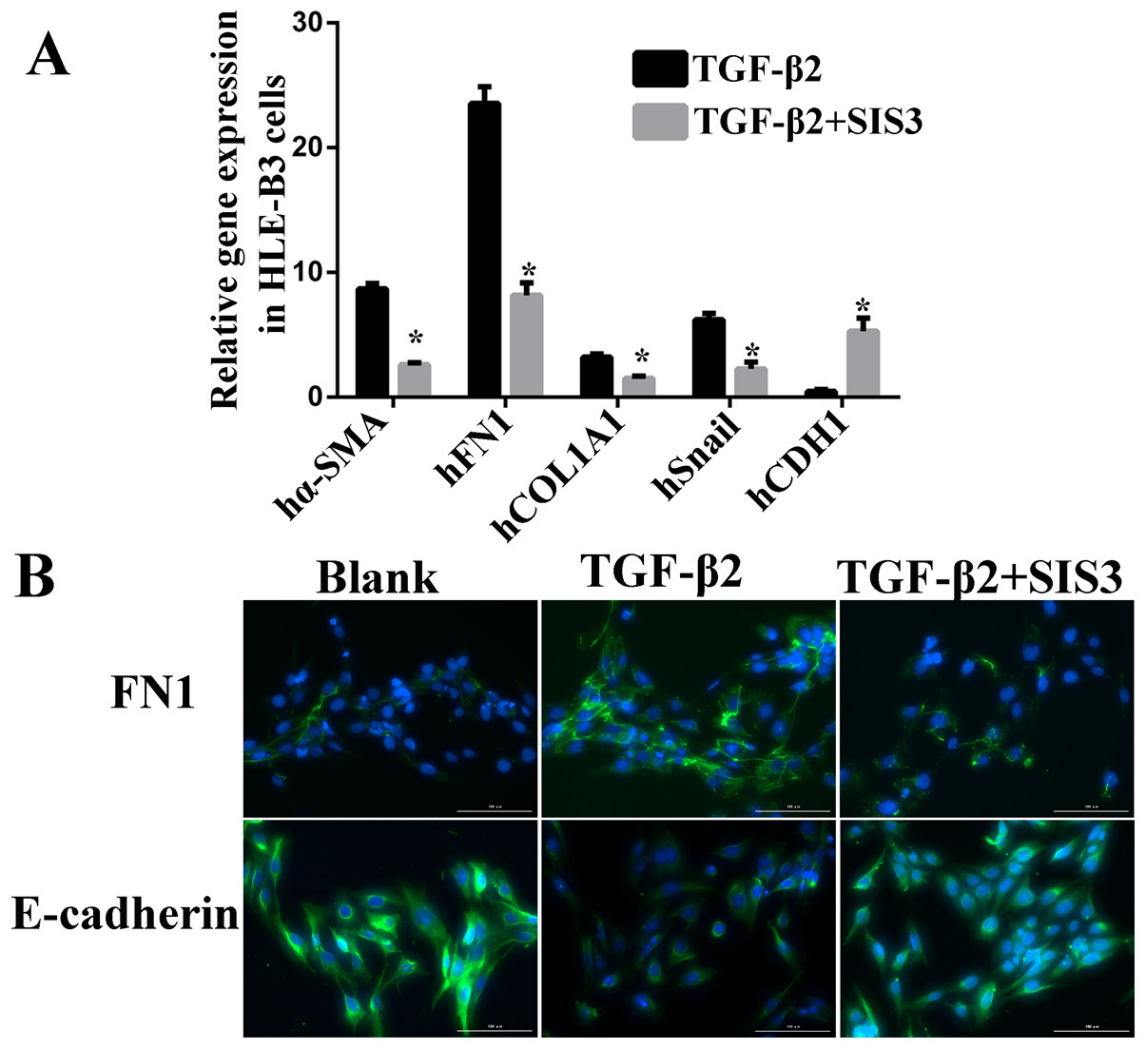
TGF-β/Smad3 pathway involved in EMT to HLE-B3 cells challenged with TGF-β2 (10 ng/ml). (A) Gene expression of EMT biomarkers inhibited by SIS3 in HLE-B3 cells. (B) Protein expression of EMT markers in HLE-B3 cells challenged with TGF-β2 (10 ng/ml) following treatment with Smad3 inhibitor SIS3. Scale bar=100 *μ*m. ^*^P<0.05 TGF-β2 + SIS3 group vs. the corresponding TGF-β2 group. TGF-β, transforming growth factor-β; Smad3, mothers against decapentaplegic 3; EMT, epithelial-mesenchymal transition; α-SMA, α-smooth muscle actin; FN1, fibronectin 1; COL1A1, collagen type 1 α1; CDH1, E-cadherin.

**Figure 3:**
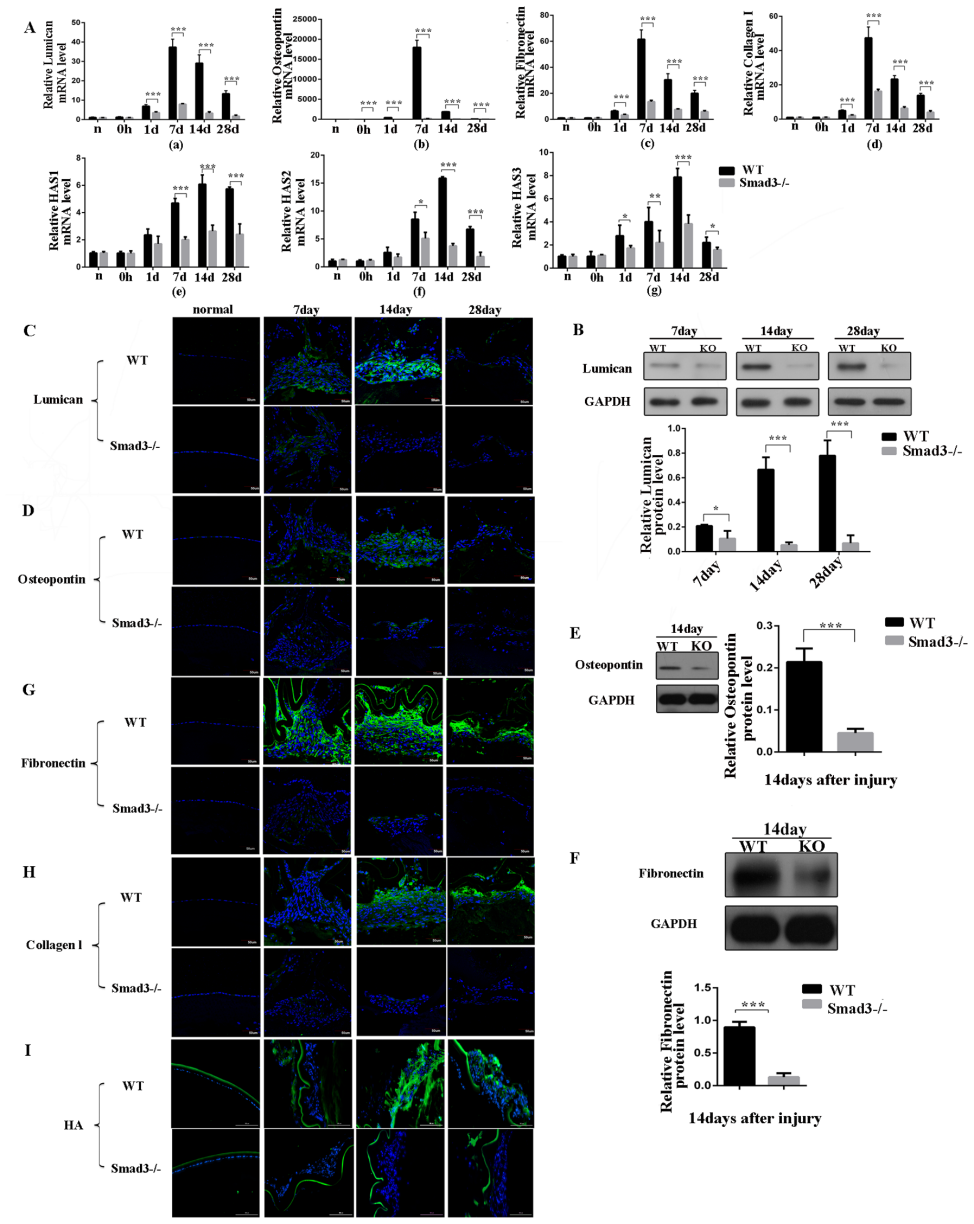
Expression levels of extracellular matrix components at different time points following anterior capsule injury. (A) mRNA expression levels of (a) lumican, (b) oesteopontin, (c) fibronectin, (d) collagen type I and (e-g) HAS in lens epithelial cells following anterior capsule injury. *In situ* protein expression levels were determined. (B) Western blot and (C) immunofluorescence of lumican; (D) immunofluorescence and (E) western blot of osteopontin; (F) western blot and (G) immunofluorescence of fibronectin. Immunofluorescence of (H) collagen type I and (I) HA in lens epithelial cells following injury. Scale bar=50 or 100 *μ*m. ^*^P<0.05, ^**^P<0.01, ^***^P<0.001. KO, knockout; WT, wild-type; Smad3, mothers against decapentaplegic 3; HAS, hyaluronan synthase; HA, hyaluronan.

